# Meta-Learning for Decoding Neural Activity Data With Noisy Labels

**DOI:** 10.3389/fncom.2022.913617

**Published:** 2022-07-06

**Authors:** Dongfang Xu, Rong Chen

**Affiliations:** Department of Diagnostic Radiology and Nuclear Medicine, University of Maryland School of Medicine, Baltimore, MD, United States

**Keywords:** neural decoding, noisy label, sample reweighting method, deep neural networks, anterior lateral motor cortex

## Abstract

In neural decoding, a behavioral variable is often generated by manual annotation and the annotated labels could contain extensive label noise, leading to poor model generalizability. Tackling the label noise problem in neural decoding can improve model generalizability and robustness. We use a deep neural network based sample reweighting method to tackle this problem. The proposed method reweights training samples by using a small and clean validation dataset to guide learning. We evaluated the sample reweighting method on simulated neural activity data and calcium imaging data of anterior lateral motor cortex. For the simulated data, the proposed method can accurately predict the behavioral variable even in the scenario that 36 percent of samples in the training dataset are mislabeled. For the anterior lateral motor cortex study, the proposed method can predict trial types with F1 score of around 0.85 even 48 percent of training samples are mislabeled.

## 1. Introduction

Neural decoding (Lee et al., 2021) centers on predicting behavioral variables based on brain-related features. That is, we aim to construct a predictive model *f*:***X***→*Y* based on a dataset **D**, where ***X*** is a vector of brain-related features. In neural decoding, ***X*** can be obtained from calcium imaging, electroencephalography, functional near-infrared spectroscopy or functional magnetic resonance imaging. The behavioral variable *Y* can be a variable representing brain states, a label for trials in trial-based analysis, or an annotation for a subject such as whether the subject progresses to Alzheimer's disease. Neural decoding enables precise neuromodulation. In precise neuromodulation, a neural decoding algorithm predicts *Y* based on neural data streams in real-time and neuromodulation parameters are tuned based on decoding results. Such closed-loop neuromodulation system could be more effective than the open-loop system.

An important step in constructing neural decoding models is sample annotation in which a label *y*_*i*_ is assigned to sample *x*_*i*_. The quality of annotation has a dramatic impact on the model generalizability which is assessed by applying the model to an independent test dataset. However, the annotation process could be subjective and the label noise problem is sometimes inevitable. The annotation quality varies across different annotators. In a study to assess the impact of noisy labels (Zhang et al., 2021), a test error rate of 0.1 was achieved by applying the Inception model to the CIFAR10 dataset, and the test error rate increased to 0.4 when 20% of labels were corrupted.

Deep neural networks (DNNs) have shown potentials to significantly improve decoding performance (Horikawa and Kamitani, 2017; Wu et al., 2021). An ideal scenario to train a DNN model is to obtain a large dataset with high-quality labels. This requires a lot of expensive expert efforts. A dataset with noisy labels is relatively easy to collect. However, most existing neural decoding methods cannot handle noisy labels. There is an urgent need to develop new deep learning methods to address the noisy label problem to improve prediction performances.

In this study, we have utilized a sample reweighting algorithm to solve the noisy label problem in neural decoding. In our neural decoding problem, we have three datasets: training, validation, and test. The training dataset is massive but labels (or annotations) are noisy. The validation dataset is small and accurately labeled. The model's performance is evaluated on a noise-free test dataset. Our goal is to address the noisy label problem in training data (noisy *Y*) to improve behavior prediction performance by utilizing a small and accurately labeled validation dataset. In the sample reweighting algorithm, training sample weights are determined to minimize the loss on the validation dataset. In practice, labeling massive data accurately is expensive and time consuming. Our neural decoding algorithm can greatly reduce the cost of this process. To the best of our knowledge, this is the first study to utilize the sample reweighting algorithm, a meta-learning method, to solve neural decoding problem.

## 2. Related Work

Noisy label problems are well studied in machine learning. DNN-based methods have been proposed to solve the label noise problems to enhance the algorithm robustness. Recent DNNs research focusing on label noise problems can be categorized into five classes (Hwanjun et al., 2022): robust architecture (Tong et al., 2015; Jacob and Ben-Reuven, 2017; Yao et al., 2019), robust regularization (Srivastava et al., 2014; Ioffe and Szegedy, 2015; Shorten and Khoshgoftaar, 2019), robust loss function (Reed et al., 2015; Zhang and Sabuncu, 2018; Lyu and Tsang, 2020), loss adjustment (Reed et al., 2015; Patrini et al., 2017; Song et al., 2019) and sample selection (Jiang et al., 2018; Wang et al., 2018). Each category has its specific properties and shows some advantages and disadvantages in handling label noise problems.

In this study, we choose loss adjustment to solve the label noise problem and improve the behavior prediction performance. It is possible to assign weights to training samples to minimize the loss on a clean unbiased validation dataset to solve the problem. The sample reweighting strategy has been well studied inclduing boosting (Freund and Schapire, 1997), hard sample mining (Malisiewicz et al., 2011), and focal loss (Lin et al., 2017). Meta-learning has been applied to improve noise robustness (Andrychowicz et al., 2016; Finn et al., 2017; Ren et al., 2018; Shu et al., 2019; Wang et al., 2020). Meta-learning centers on learning to learn better and is an active topic in machine learning. Ren et al. have proposed a novel meta-learning algorithm (Ren et al., 2018) that learns to assign weights to training samples based on their gradient directions. This method improves the training objective by a weighted loss rather than an average loss and is an example of meta-learning. With meta-learning, the trained network easily adapts to various types of data and label noise. Though this method has been explored in recent meta-learning research (Ravi and Larochelle, 2017; Ren et al., 2018), our study is the first to apply sample reweighting meta-learning algorithm to neural activity data.

## 3. Methods

In this section, we describe the sample reweighting method and a baseline method to solve the noisy label problem. The sample reweighting algorithm used in this paper is DNN-based and proposed in Ren et al. (2018). We apply it to neural decoding. The training dataset is denoted as {(*x*_*i*_, *y*_*i*_), 1 ≤ *i* ≤ *N*}, where *x*_*i*_ denotes the *i*^*th*^ sample, *y*_*i*_ is the label corresponding to *x*_*i*_, and *N* is the sample size of the training dataset. *y*_*i*_ is noisy. The validation dataset is relatively small but accurately labeled, which is denoted as {$\left(x_{i}^{\upsilon },y_{i}^{\upsilon }\right),1\leq i\leq M$}, where $x_{i}^{\upsilon }$ denotes the *i*^*th*^ sample, $y_{i}^{\upsilon }$ denotes the label corresponding to $x_{i}^{\upsilon }$, and *M* is the sample size of the validation dataset. We use θ to denote the parameters of neural network model.

### 3.1. Sample Reweighting Method

Our goal is to reweight training samples and minimize a weighted loss $\overset{N}{\underset{i=1}{\sum }}\omega_{i}f_{i}\left(\theta \right)$. ω is calculated based on the validation performance, i.e. $\omega^{*}=\underset{\omega ,\;\omega \geq 0}{\arg\min}\frac{1}{M}\overset{M}{\underset{i=1}{\sum }}f_{i}^{\upsilon }\left(\theta^{*}\left(\omega \right)\right)$. For DNNs, stochastic gradient descent (SGD) is used to optimize loss function and the model parameters are adjusted according the descent direction. SGD can be formulated as follows:


(1) $$\begin{array}{l} \theta_{t+1}=\theta_{t}-\alpha \nabla \left(\frac{1}{n}\overset{n}{\underset{i=1}{\sum }}f_{i}\left(\theta_{t}\right)\right), \end{array}$$

where α is the step size. According to Koh and Liang (2017), we consider perturbing the weight by ϵ_*i*_ for each training sample in the mini-batch,


(2) $$\begin{array}{l} f_{i,\epsilon }\left(\theta \right)=\epsilon_{i}f_{i}\left(\theta \right), \end{array}$$

(3) $$\begin{array}{l} {\hat{\theta }}_{t+1}(\epsilon )=\theta_{t}-\alpha \nabla \sum_{i=1}^{n}f_{i,\epsilon }(\theta ){|}_{\theta =\theta_{t}}, \end{array}$$

By minimizing the validation loss *f*^υ^ locally at step *t*, we can obtain an optimal ϵ^*^ as follows:


(4) $$\begin{array}{l} \epsilon_{t}^{*}=\underset{\epsilon }{\arg\;\min}\frac{1}{M}\sum_{i=1}^{M}f_{i}^{\upsilon }(\theta_{t+1}(\epsilon )), \end{array}$$

Taking a single gradient descent step on a mini-batch of validation dataset and rectify the output, we obtain the ${\tilde{\omega }}_{i,t}$, which is calculated as follows:


(5) $$\begin{array}{l} \mu_{i,t}=-\eta \frac{\partial \frac{1}{m}\sum_{j=1}^{m}f_{j}^{\upsilon }(\theta_{t+1}(\epsilon ))}{\partial \epsilon_{i,t}}{|}_{\epsilon_{i,t}=0}, \end{array}$$

(6) $$\begin{array}{l} {\tilde{\omega }}_{i,t}=max\left(\mu_{i,t},0\right), \end{array}$$

We normalize the weights of all samples so that their sum is one. This is a hard constraint and can be seen as follows:


(7) $$\begin{array}{l} \omega_{i,t}=\frac{{\tilde{\omega }}_{i,t}}{\sum {\tilde{\omega }}_{j,t}+\delta \left(\sum {\tilde{\omega }}_{j,t}\right)}, \end{array}$$

The normalization can prevent the degenerate case and cancel the meta-learning rate parameter η (Ren et al., 2018). The process is listed in Algorithm 1.

**Algorithm 1:** Sample Reweighting Algorithm


**Require:**
θ_0_, *m, n*, training data Ω_*f*_, validation data Ω_*g*_
**Ensure:**
θ_*T*_
1: **for** t = 0...T-1 **do**
2: Sample a mini-batch from Ω_*f*_: {*X*_*f*_, *y*_*f*_}
3: Sample a mini-batch from Ω_*g*_: {*X*_*g*_, *y*_*g*_}
4: Train a model and predict: ŷ_*f*_ = Forward(*X*_*f*_, *y*_*f*_, θ_*t*_)
5: Calculate loss: *l*_*f*_ = $\overset{n}{\underset{i=1}{\sum }}\epsilon_{i}C\left(y_{f,i},{ŷ}_{f,i}\right)$; (ϵ: to perturb the weighting)
6: Update model parameters: ${\hat{\theta }}_{t}$ = θ_*t*_ - α∇θ_*t*_;
7: Predict samples in the validation dataset: ŷ_*g*_ = Forward($X_{g},y_{g},{\hat{\theta }}_{t}$)
8: Calculate validation loss: *l*_*g*_ = $\frac{1}{m}\overset{m}{\underset{i=1}{\sum }}C\left(y_{g,i},{ŷ}_{g,i}\right)$
9: Rectify weighting: $\hat{\omega }$ = max(-∇ϵ, 0)
10: Normalize weighting: ω = $\frac{{\tilde{\omega }}_{i,t}}{\sum {\tilde{\omega }}_{j,t}+\delta \left(\sum {\tilde{\omega }}_{j,t}\right)}$
11: Calculate weighted training loss: ${\hat{l}}_{f}$ = $\overset{n}{\underset{i=1}{\sum }}\omega_{i}C\left(y_{i},{ŷ}_{f,i}\right)$
12: Update model parameter: θ_*t*+1_ = OptimizerStep(θ_*t*_, ∇θ_*t*_); (∇θ_*t*_ = BackwardAD(${\hat{l}}_{f},\theta_{t}$))
13: **end for**

The DNN for the sample reweighting method has an input layer, two hidden layers and an output layer. Although a single sufficiently large hidden layer is adequate to approximate most functions, this design is inefficient compared to designs with more layers. The two (or more) hidden layers architecture is more efficient (Reed and MarksII, 1999). There are some empirically-derived rules to determine the number of nodes in each layer: the number of hidden nodes should be 2/3 the size of the input layer, plus the size of the output layer, and less than twice the size of the input layer. In our study, the sizes of the input and output layers for the simulated dataset were 80 and 2, respectively; and they were 91 and 2 for the ALM dataset, respectively. Therefore, our DNN had two hidden layers, and the two hidden layers contained 128 and 64 nodes, respectively. The activation functions in the two hidden layers are the rectified linear unit (ReLU) functions. The loss function is the cross entropy loss, and the optimizer is the Adam algorithm. For model learning, there exist some parameters: learning rate, batch size and epoch size. The learning rate and epoch size are tuned according to the weighted loss. The batch size is set as the same as the sample size of validation dataset because the sample reweighing method updates weights by taking a single gradient descent step on a mini-batch of validation dataset. Model convergence is determined according to the weighted loss, when it decreases very slowly or becomes flat.

### 3.2. Baseline Method

Two baseline methods are used for the comparison purpose. Sample reweighting is not used in the baseline methods. In the first baseline method (baseline 1), we merge the training and validation data. Model parameters are tuned according to the performance based on the merged dataset. In the second baseline method (baseline 2), we train the DNN model based on the validation dataset only. The DNNs of these baseline methods contain one input layer, two hidden layers and one output layer. The input layer includes all features. The method applies a linear transformation to the input features and transfers the transformed features to the first hidden layer. The first hidden layer contains 128 neurons and the activation function is ReLU. The second hidden layer contains 64 neurons and the activation function is also the ReLU. The loss function is the cross entropy loss. The optimizer is the Adam algorithm.

## 4. Experiments and Results

We evaluated the sample reweighting method based on the simulated data and calcium imaging data of anterior lateral motor cortex (ALM) (Li et al., 2015). For a study, the training data had label noises, the validation data had few mislabeled samples or clean, and the test data were clean. An algorithm's performance is evaluated based on the test dataset. The prediction quality metrics are the F1 score and balanced accuracy.

### 4.1. Simulated Data

The neuron model in the simulated data was integrate-and-fire neurons with additive noise. Our simulation included 80 neurons. There were two groups of neurons: group A and group B. Each group had 40 neurons. Neurons in group A were activated by a stimulus and neurons in group B received synaptic inputs from two or three neurons in group A. Neurons in groups A and B formed a two-layer feed-forward neural network. The feature vector included neural activities of groups A and B. Its dimension was 80. The behavioral variable *y* is a non-linear function of the average population activity of group B. Let *r*_*B*_ denote the average population activity of group B. If *sin*(π·*r*_*B*_)>0.85, *y* = 1; otherwise, *y* = 0.

The simulated data consisted of training, validation, and test data. Our task was to predict *y*. Each training dataset had 600 samples. The class distribution was imbalanced. The ratio of the number of samples of classes 0 and 1 was around 3:1. For the training dataset, we simulated noisy labeling by randomly shuffling a portion of *y*. For example, the portion parameter was 80% representing a case that 0.8 × 600 = 480 data points of *y* were randomly shuffled. We generated five training datasets with different noise levels. For these five training datasets, the portion parameters are 100, 80, 60, 40, and 20%. Table 1 summarized the training data. These datasets were referred to as annotators 1–5. The label noise levels of training samples after shuffling were listed in the last column of Table 1.

**Table 1:** The training dataset of the simulated data study.

**Annotator**	**Sample**	**Sample size**	**Sample size**	**Portion**	**Noise**
	**Size**	**(Label 0)**	**(Label 1)**	**Parameter(%)**	**Level(%)**
1	600	464	136	100	36.3
2	600	460	140	80	29.6
3	600	456	144	60	23.0
4	600	460	140	40	13.0
5	600	461	139	20	7.3

The validation dataset was accurately labeled with portion parameter = 10%. Most of samples in the validation dataset were labeled correctly. We built a relatively big validation dataset with 600 samples. The sample sizes for classes 0 and 1 were 457 and 143, respectively. However, in our experiments, we might only use a small portion of validation dataset (for example, 20 samples from the validation dataset). We created this large validation dataset in order to assess the algorithm's performance relative to the sample size of the validation dataset. In real-world applications, we always prefer a small validation dataset due to the cost of obtaining high quality annotations. The test dataset was clean and without noise. The sample size of test dataset was 600, and the samples sizes for classes 0 and 1 were 449 and 151, respectively. Both the validation and test datasets were imbalanced.

#### 4.1.1. Sample Reweighting Is Effective

**Figure 1:**
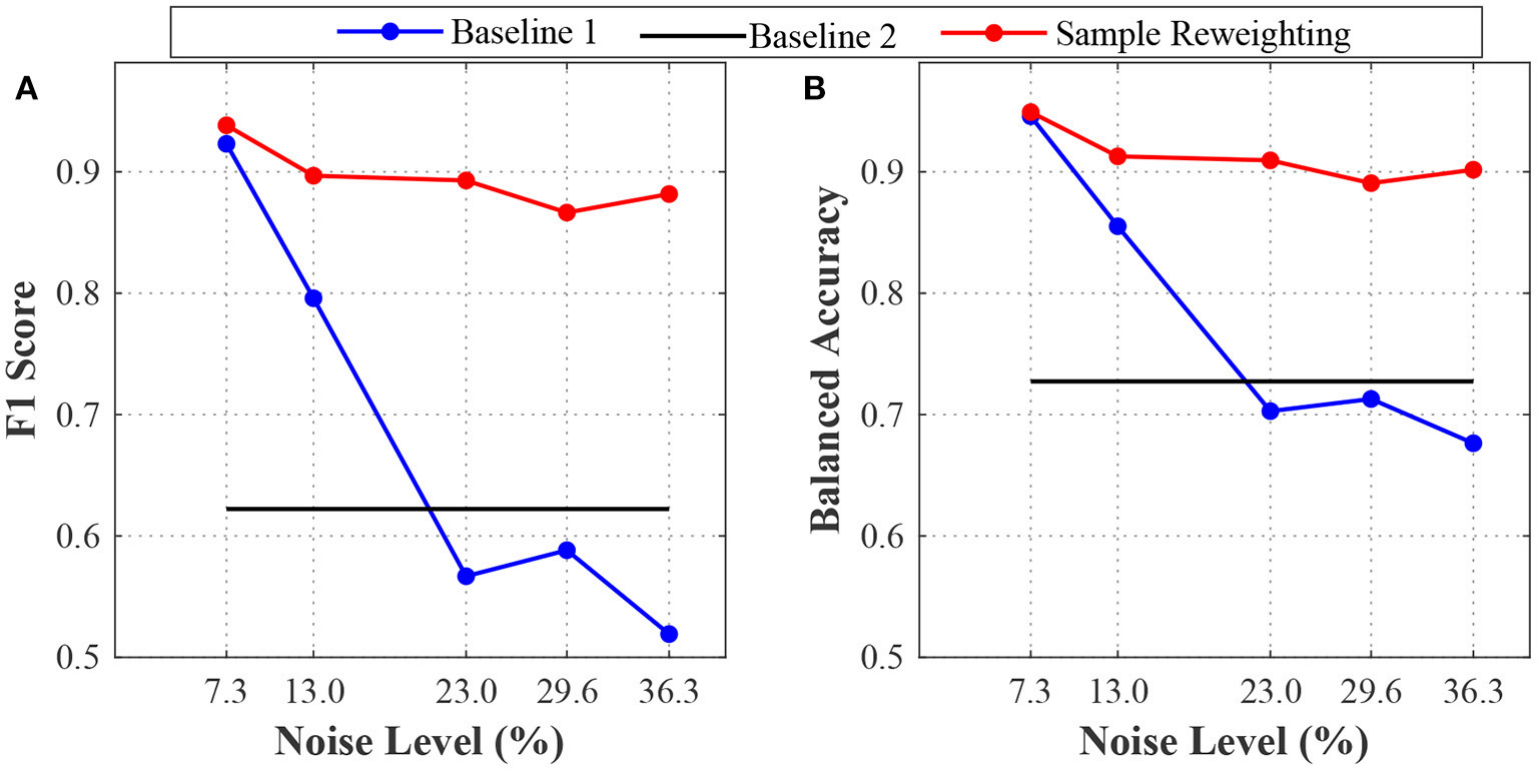
The model's performances on the simulation datasets with different label noise levels. **(A)** is the F1 scores of baselines 1 and 2 and sample reweighting method. **(B)** is the balanced accuracies of baseline 1 and 2 and sample reweighting method.

The first sub-experiment aimed to assess model generalizability under different noise levels for the sample reweighting and baseline methods. For the sample reweighting method, we used a very small validation dataset (20 samples) to guide training. For the baseline and sample reweighting methods, the model's performances for different noise levels are shown in Figure 1. For baseline 1, when the noise level increased, the model performances (F1 score and balanced accuracy) decreased significantly. The F1 score and balanced accuracy of the sample reweighting method remained stable across noise levels. At 7.3% noise level, the F1 score and balanced accuracy were greater than 0.9. When the noise level was 36.3%, both the F1 score and balanced accuracy of the sample reweighting method were still around 0.9. When the noise level increased from 7.3 to 36.3%, the F1 score of sample reweighting method dropped less than 0.1, whereas that of baseline 1 dropped around 0.4. When the noise level increased from 7.3 to 36.3%, the balanced accuracy of the sample reweighting method dropped less than 0.05, whereas that of the baseline 1 dropped more than 0.2. The sample reweighting method obtained better performances than baseline 2. Because baseline 2 didn't use training data, the method's performance was constant across training data noise levels. This sub-experiment demonstrated the effectiveness of the sample reweighting method.

**Figure 2:**
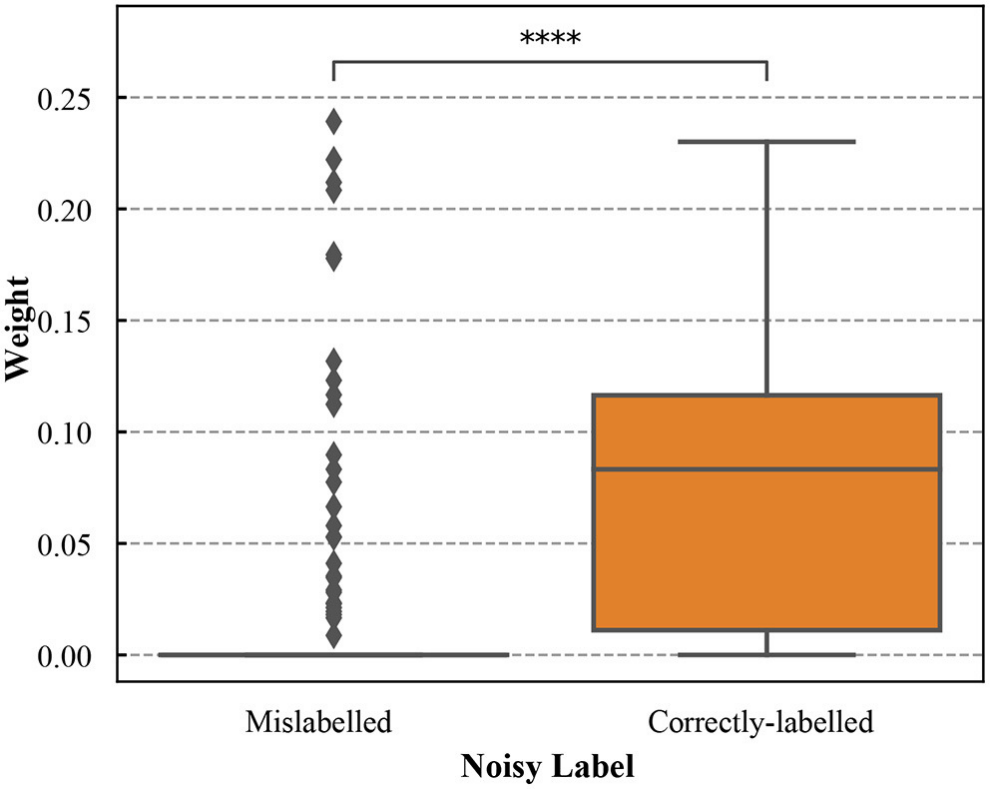
The boxplots of weights for the correctly-labeled and mislabeled samples in the simulation training dataset with noisy labels (the noise level is 36.3%). **** denotes that weights of the correctly-labeled samples were significantly different from those of mislabeled samples (*p* < 0.0001).

To illustrate how the samples were reweighted after meta-learning, the sample weight distribution for the training dataset was shown in Figure 2. The correctly-labeled samples in the training dataset were up-weighted. Most of the mislabeled samples (86.70%) had weight = 0. We conducted the Wilcoxon rank-sum test to compare the weights between correctly-labeled and mislabeled samples. Weights of the correctly-labeled samples were significantly different from those of mislabeled samples (*p* < 0.0001).

#### 4.1.2. The Sample Reweighting Method Is Robust to the Validation Sample Size

**Figure 3:**
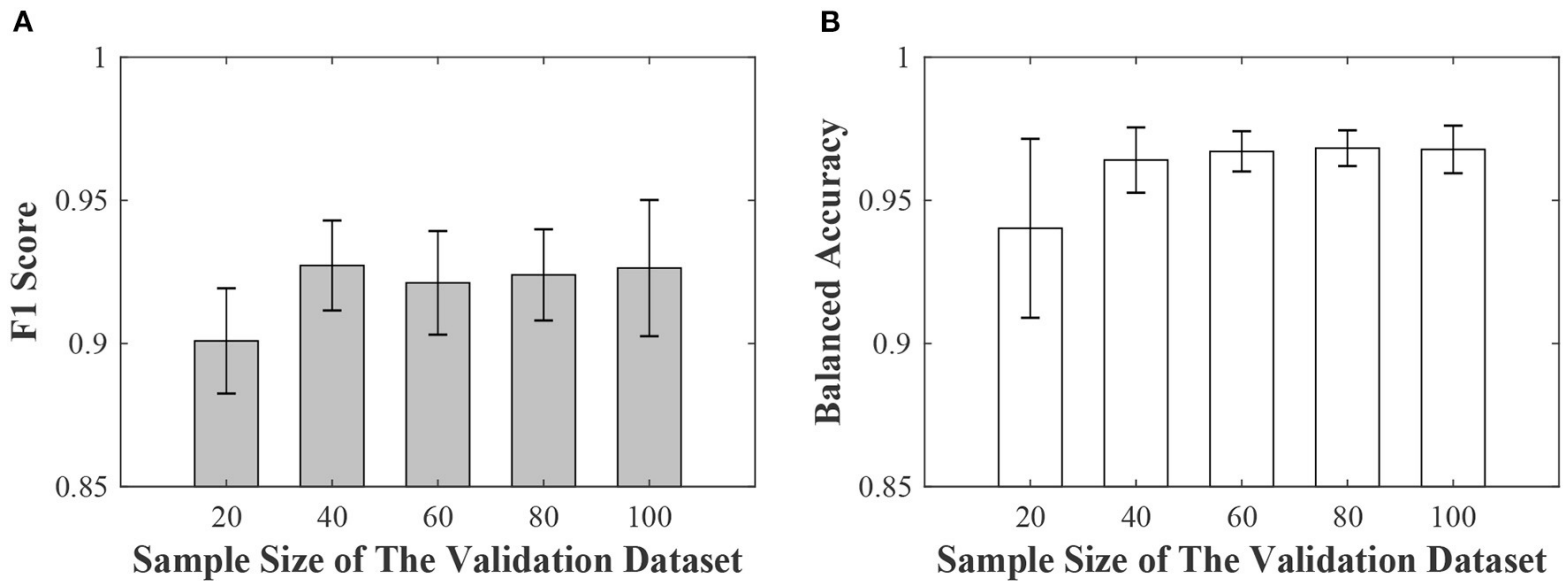
The effects of sample size of validation data. **(A)** is the averages (the gray bars) and the standard deviations (the black line) of the F1 score. **(B)** is the averages (the white bars) and the standard deviations (the black line) of the balanced accuracy.

Our second sub-experiment aimed to assess the effects of sample size of validation dataset. In this sub-experiment, the noise level of the training datasets was 36.3%, and the sample sizes of the validation dataset were 20, 40, 60, 80 and 100, respectively. For a given validation sample size, we constructed five validation datasets by randomly sampling the original dataset to reflect sampling variability. We calculated the means and standard deviations. The results (mean ± standard deviation) were shown in Figure 3. When the sample size of validation was 20, the F1 score and balanced accuracy were still greater than 0.9. The model's performance was relatively stable for different validation sample sizes. We didn't observe a significant drop in the performance curve.

#### 4.1.3. The Sample Reweighting Method Can Handle Training Data With Mixed Annotation Quality

**Figure 4:**
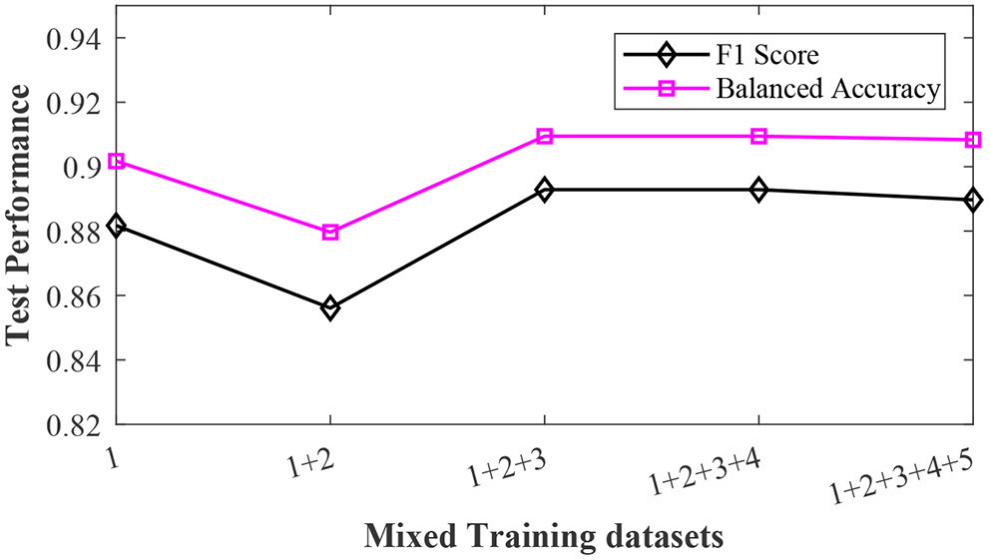
The effects of the mixed training datasets. The pink squares and black diamonds denote the balanced accuracy and F1 score, respectively.

In many real-world applications, we want to pool datasets with different annotation qualities to increase statistical power. For example, we want to merge two training datasets that are labeled by annotators with different experience levels. In the third sub-experiment, we merged the training datasets with different noise levels together and then test the sample reweighting method on the mixed training dataset. The results are shown in Figure 4. Although the training dataset contained data generated with different label noise levels, the sample reweighting method achieved good test performances. The balanced accuracy was always greater than 0.88 and the F1 score was greater than 0.84.

### 4.2. The Anterior Lateral Motor Cortex Study

**Figure 5:**
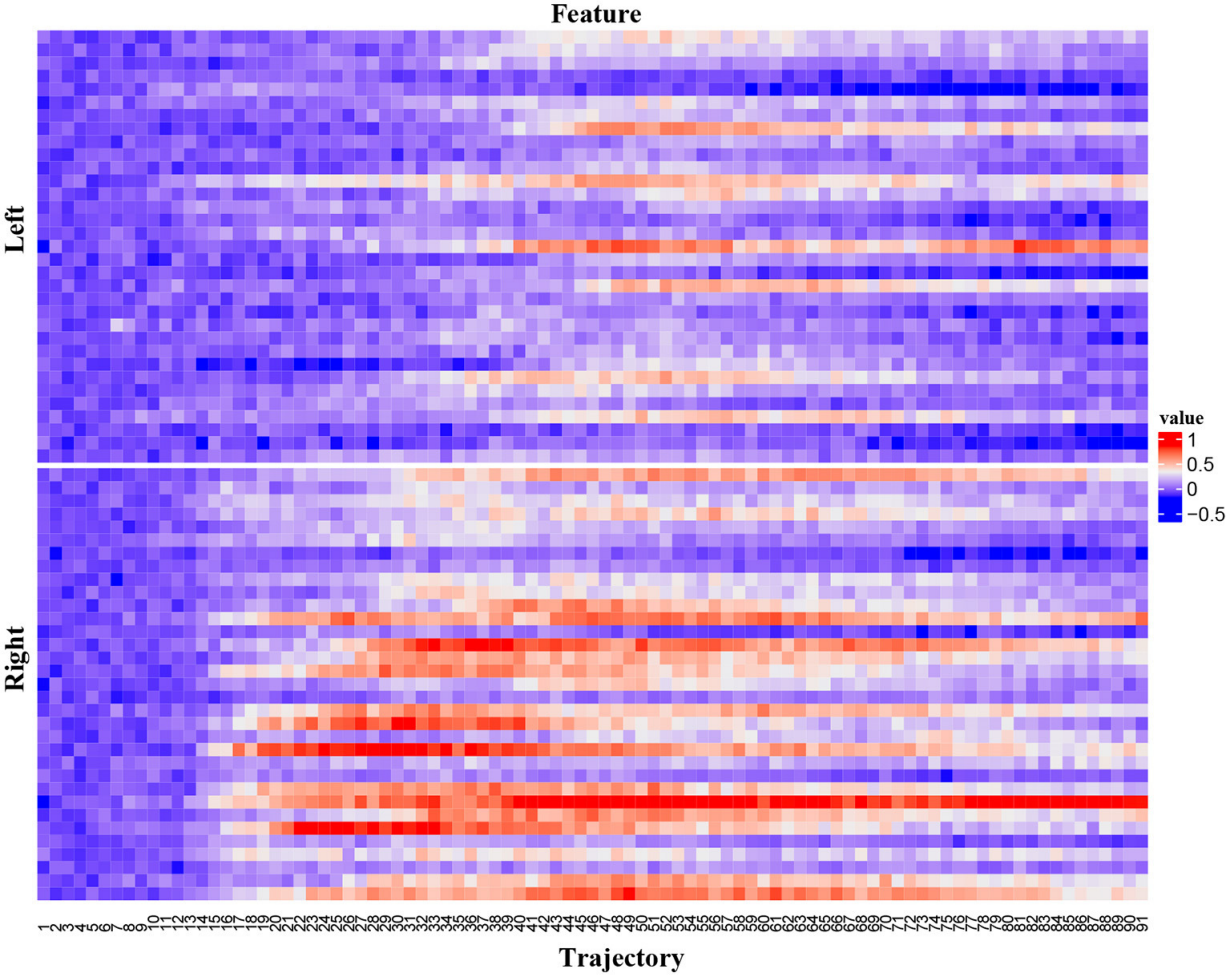
The ALM data. The upper panel is for trial type “left” and the bottom panel is for trial type “right”.

In mice, activities in the anterior lateral motor cortex (ALM) predict movements. We reanalyzed two-photon calcium imaging data of ALM (Li et al., 2015). In this dataset, mice underwent a whisker-based object location discrimination task. A trial included three epochs: sample, delay, and response. A pole in the anterior or posterior position was presented during the sample epoch. During the response epoch, mice reported the perceived pole position by licking right for posterior or licking left for anterior. Two-photon calcium imaging data of neurons in the left ALM were acquired. We analyzed calcium imaging from a single subject (subject an019). This subject had 18 imaging sessions and the number of trials of a session was in [48, 67]. Each trial had 91 time points. For a time point, we calculated the ensemble neural activity and used this trial trajectory as the feature vector. The dimension of this feature vector was 91. Figure 5 is an example of original ALM data. It depicts 66 trial trajectories of session an019-2013-08-20-275. The x-axis is the trial trajectory. All the original ALM data were preprocessed by zero-mean normalization. The label variable is the trial type (left or right).

We divided the original ALM dataset into training, validation, and test datasets. The description of the ALM study is in Table 2. The test dataset included two sessions (an019-2013-08-16-370 and an019-2013-08-19-410) because these two sessions had high signal-to-noise ratios. Another high signal-to-noise ratio session, an019-2013-08-20-275, was selected as the validation dataset. Other sessions were used as the training data. We made five copies of the original training dataset and then added label noises into each copy to obtain five training datasets with different label noise levels. For these five copies, the portion parameters are 100, 80, 60, 40, and 20%. The label noise levels of each copied training dataset after shuffling were listed in the last column of Table 2. The validation dataset was small (66 samples) and clean. It had 33 samples for each trial type. The test dataset was clean and had 103 samples, and the sizes of samples belonging to the two trial types were 53 and 50, respectively.

**Table 2:** The ALM study.

**No**.	**Sample**	**Sample size**	**Sample size**	**Portion**	**Noise**
	**size**	**(Label 0)**	**(Label 1)**	**Parameter**	**Level**
Training 1	853	446	407	100%	52.3%
Training 2	853	446	407	80%	41.3%
Training 3	853	446	407	60%	31.0%
Training 4	853	446	407	40%	21.1%
Training 5	853	446	407	20%	9.9%
Validation	66	33	33	0 (Clean)	0
Test	103	53	50	0 (Clean)	0

**Figure 6:**
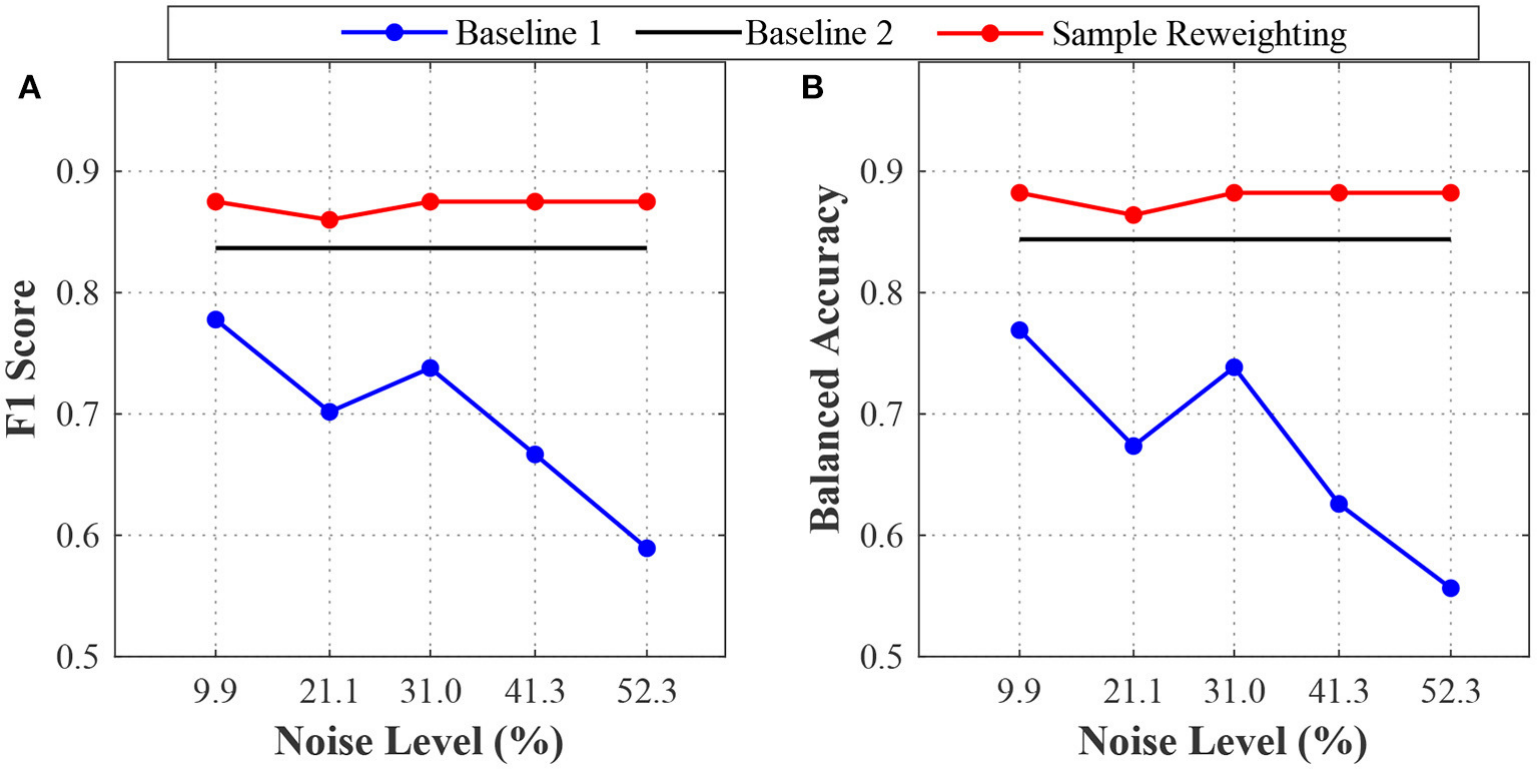
The test performances on the ALM data. **(A)** is the F1 scores of baselines 1 and 2 and sample reweighting method. **(B)** is the balanced accuracies of the baseline 1 and 2 and sample reweighting method.

We evaluated the sample reweighting method and baseline methods based on the ALM dataset. The results are depicted in Figure 6. When the noise level increased, the sample reweighting method and baseline 1 performed quite differently. When the noise level was 9.9%, the F1 scores and balanced accuracy of the sample reweighting method were about 0.9, while they were smaller than 0.8 for baseline 1. Even when the noise level was 52.3%, the sample reweighting method still achieved good performance and the F1 score and balanced accuracy were about 0.9, while they were smaller than 0.6 for baseline 1. When the noise level increased from 9.9 to 52.3%, the F1 score and balanced accuracy of the sample reweighting method dropped less than 0.05, whereas they dropped nearly about 0.2 for baseline 1. The sample reweighting method obtained better performances than baseline 2, as shown in Figure 6.

**Figure 7:**
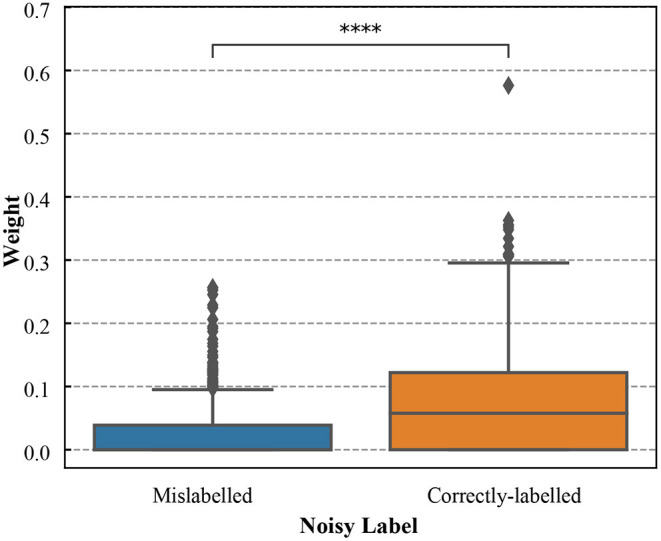
The boxplots of weights for the correctly-labeled and mislabeled samples in ALM training dataset (the noise level is 52.3%). **** denotes that weights of the correctly-labeled samples were significantly different from those of mislabeled samples (*p* < 0.0001).

The weight distribution (after meta-learning) for the training data was shown in Figure 7. The correctly-labeled samples in training dataset were up-weighted while the mislabeled samples were down-weighted. There was a significant difference between weights of the correctly-labeled and those of mislabeled samples (*p* < 0.0001, the Wilcoxon rank-sum test).

## 5. Discussion

In this study, we propose to utilize a sample reweighting algorithm that learns to assign weights to training data to solve the label noise problem in neural decoding. The sample weights are determined by minimizing the loss on a clean validation set. The sample reweighting method requires a training dataset that is massive but coarsely labeled and a validation dataset that is small but accurately labeled. In many neural decoding applications, labeling massive data accurately is expensive. The sample reweighting method can improve the neural decoding performances by guiding the massive coarsely labeled training data with a small clean validation dataset. This can greatly reduce the annotation cost. To evaluate this method, we have designed two experiments based on the simulated neural signal data and ALM data. Each dataset contains several training datasets with different label noise levels, one validation dataset with few or without label noises and one test dataset without label noises. Also, there is class imbalance in the simulated dataset.

Based on the simulated neural signal data, we have conducted a series of experiments. The first sub-experiment is performance analysis of sample reweighting algorithm on training datasets with different noise levels. The results have shown that the sample reweighting method achieved better performances (higher F1 score and balanced accuracy) than the baseline methods. At any noise level, the F1 score and balanced accuracy based of the sample reweighting method were greater than 0.85. As the noise level increased, the performances of baseline 1 decreased significantly. This experiment has illustrated that the sample reweighting method is effective in dealing with noisy labels. Secondly, we assessed the impact of the sample size of the validation dataset. It is expensive to label a big dataset accurately. We need to balance the sample size of validation dataset and model performance. The results are shown in Figure 3. When the validation sample size increased from 20 to 40, both F1 score and balanced accuracy increased. When the validation sample size increased from 40 to 100, the balanced accuracy increased non-significantly. The averages of F1 score and balanced accuracy are greater than 0.9 when validation sample size is 20. Thirdly, we have studied the mixed label quality problem to determine whether the sample reweighting method works well when we pool training data with different noise levels. The results show that the sample reweighting method works well.

To evaluate the effectiveness of sample reweighting on real-world application, we applied the sample reweighting method to the ALM dataset (Li et al., 2015). Based on the original dataset, we have constructed new training datasets with different label noise levels, and generated five training datasets which were coarsely labeled and a validation dataset which was small but accurately labeled. The results are shown in Figure 6. We found that the sample reweighting method was effective and had better performances than the baseline methods. At any noise level, the F1 score and balanced accuracy of the sample reweighting method were greater than 0.85.

To understand the impact of the training dataset, we also included a baseline method (baseline 2) that trains a DNN based on the validation dataset alone. For both simulated data and the ALM dataset, we found the sample reweighting method that uses validation data to guide training obtained better performances than baseline 2. We found that there was a significance difference between weights for the correctly-labeled and those of mislabeled samples (*p* < 0.0001, the Wilcoxon rank-sum test). The correctly-labeled samples in training data were up-weighted while the mislabeled samples were down-weighted. Collectively, these results demonstrated that the sample reweighting method used the information from both training and validation datasets and effectively down-weighted mislabeled samples in the training data based on the validation data.

In this study, we have adopted a sample reweighting method by reweighting training samples to train models to solve the label noise problem in neural decoding. As the sample reweighting algorithm is based on DNNs, there are some parameters to tune (such as learning rate, batch size and epoch size). This is a common problem in deep learning. Our future work will explore using automatic machine learning methods to tune these parameters. In this paper, we used multilayer perceptrons for features in vector form. The proposed method can also be used for other neural network architectures such as convolutional neural networks. In the future, we will apply the proposed method to neuroimaging data, sequence data, or graph data.

## Data Availability Statement

The original contributions presented in the study are included in the article/supplementary material, further inquiries can be directed to the corresponding author.

## Author Contributions

RC designed the study and implemented the models. DX analyzed the model and conducted experiments. DX and RC wrote the manuscript. Both authors contributed to the article and approved the submitted version.

## Funding

This work was supported by the NIH NINDS and the BRAIN Initiative (R01NS110421) and NIH NIDA (UG3DA053802).

## Conflict of Interest

The authors declare that the research was conducted in the absence of any commercial or financial relationships that could be construed as a potential conflict of interest.

## Publisher's Note

All claims expressed in this article are solely those of the authors and do not necessarily represent those of their affiliated organizations, or those of the publisher, the editors and the reviewers. Any product that may be evaluated in this article, or claim that may be made by its manufacturer, is not guaranteed or endorsed by the publisher.
